# Longitudinal transcriptomic characterization of the immune response to acute hepatitis C virus infection in patients with spontaneous viral clearance

**DOI:** 10.1371/journal.ppat.1007290

**Published:** 2018-09-17

**Authors:** Brad R. Rosenberg, Marion Depla, Catherine A. Freije, Denis Gaucher, Sabrina Mazouz, Maude Boisvert, Nathalie Bédard, Julie Bruneau, Charles M. Rice, Naglaa H. Shoukry

**Affiliations:** 1 Department of Microbiology, Icahn School of Medicine at Mount Sinai, New York, NY, United States of America; 2 Program in Immunogenomics, The Rockefeller University, New York, NY, United States of America; 3 Centre de Recherche du Centre Hospitalier de l’Université de Montréal (CRCHUM), Montréal, QC, Canada; 4 Departement de microbiologie, infectiologie et immunologie, Université de Montréal, Montréal, QC, Canada; 5 Département de médecine familiale et de médecine d’urgence, Université de Montréal, Montréal, QC, Canada; 6 Laboratory of Virology and Infectious Disease, The Rockefeller University, New York, NY, United States of America; 7 Center for the Study of Hepatitis C, The Rockefeller University, New York, NY, United States of America; 8 Département de médecine, Université de Montréal, Montréal, QC, Canada; The Rockefeller University, UNITED STATES

## Abstract

Most individuals exposed to hepatitis C virus (HCV) become persistently infected while a minority spontaneously eliminate the virus. Although early immune events influence infection outcome, the cellular composition, molecular effectors, and timeframe of the host response active shortly after viral exposure remain incompletely understood. Employing specimens collected from people who inject drugs (PWID) with high risk of HCV exposure, we utilized RNA-Seq and blood transcriptome module (BTM) analysis to characterize immune function in peripheral blood mononuclear cells (PBMC) before, during, and after acute HCV infection resulting in spontaneous resolution. Our results provide a detailed description of innate immune programs active in peripheral blood during acute HCV infection, which include prominent type I interferon and inflammatory signatures. Innate immune gene expression rapidly returns to pre-infection levels upon viral clearance. Comparative analyses using peripheral blood gene expression profiles from other viral and vaccine studies demonstrate similarities in the immune responses to acute HCV and flaviviruses. Of note, both acute dengue virus (DENV) infection and acute HCV infection elicit similar innate antiviral signatures. However, while transient in DENV infection, this signature was sustained for many weeks in the response to HCV. These results represent the first longitudinal transcriptomic characterization of human immune function in PBMC during acute HCV infection and identify several dynamically regulated features of the complex response to natural HCV exposure.

## Author summary

Hepatitis C virus (HCV) is a leading cause of liver disease, with an estimated 71 million people infected worldwide. Following exposure, a subset of individuals spontaneously clears the virus while a majority progress to chronic infection. The immune functions active during the initial, acute infection by HCV are not well understood, due in part to difficulties associated with studying the early stages of this disease in humans. Most individuals acutely infected with HCV are asymptomatic and do not initially seek medical care, which presents challenges in conducting prospective longitudinal studies. Here, by employing specimens from a rare research cohort of individuals sampled before infection, during infection, and after spontaneous viral clearance, we use RNA-Seq to characterize the early immune processes active in acute HCV. Our analysis identified a robust innate antiviral gene signature that corresponds with HCV viremia. In addition, comparisons to other immune transcriptomics datasets demonstrated that the immune response to acute HCV shares many features with responses to flaviviruses. These results offer a detailed longitudinal description of immune function active during the spontaneous resolution of acute HCV infection and provide insight into the early events that may contribute to viral clearance.

## Introduction

Despite the recent breakthrough of highly effective direct acting antiviral therapies, hepatitis C virus (HCV) remains a significant public health threat. New infections, especially among people who inject drugs (PWID), are likely to increase in the absence of a prophylactic vaccine [1]. Effective vaccine development is hampered by our limited understanding of how protective immunity is established in the acute stages of natural infections. Acute HCV infection has two dichotomous outcomes, spontaneous resolution (~25% of infections) or chronic infection (~75% of infections) [2]. Immune functions following viral exposure remain incompletely understood due in part to the limited availability of paired pre-infection and longitudinal acute infection research samples from recently exposed, largely asymptomatic individuals.

Previous work has established roles for innate and adaptive immunity in the host response to acute HCV. Genetic polymorphisms at the IFNL3/4 locus, NK cell activity, and dendritic cell function influence infection outcomes [3, 4]. Effective adaptive immunity is also essential for HCV clearance. HCV-specific CD4 and CD8 T cell responses are induced in most acutely infected individuals irrespective of outcome. However, failure to sustain CD4 T cell responses is associated with viral persistence, which in turn leads to CD8 T cell dysregulation and exhaustion [3, 4]. The role of B cells in acute HCV is less clear. Although not consistent across all studies, anti-HCV neutralizing antibodies have been associated with spontaneous clearance during primary and secondary infections, suggesting that they may contribute to long-term protective immunity [5]. Kinetics and crosstalk between these innate and adaptive responses remain incompletely defined.

Systems-level transcriptomic methods have emerged as powerful tools for profiling human immune responses [6]. Examining peripheral blood transcriptome data has provided integrated maps of host response dynamics following vaccination or infection, and the associated interplay of innate and adaptive immune components [7–10]. Studies of the responses to yellow fever and influenza vaccines have identified shared gene expression signatures associated with strong antibody responses [7, 9–12]. Related studies of influenza [13] and hepatitis B virus (HBV) vaccines [14] have underscored the role of baseline inflammation and host factors in determining the outcome of vaccination. Although challenging due to logistical demands and interindividual variation, analogous methods have also been successful in characterizing the human immune response to “real world” acute infections by pathogens such as dengue virus (DENV) [15]. We reasoned that similar transcriptomic approaches, with the potential to extract large amounts of data from relatively limited sample material would be useful in characterizing the response to acute HCV.

The first microarray studies of acute HCV infection performed on serial liver biopsies from a limited number of chimpanzees showed that innate immune responses are rapidly induced in the liver irrespective of infection outcome [16, 17]. Spontaneous HCV clearance was associated with upregulation of genes linked to CD4 T cells and lymphocyte migration to the liver [16, 17]. More recent transcriptomic studies of HCV-specific T cells indicate that metabolic dysregulation during acute infection may influence the outcome of the antiviral T cell response [18]. Transcriptome analysis in human livers demonstrated elevated IFNγ–stimulated gene expression in acute infection, but elevated IFNα–stimulated gene expression during chronic infection [19]. Additional microarray studies described elevated interferon stimulated gene (ISG) expression in peripheral immune cells during chronic HCV [20].

Despite our understanding of certain aspects of the host response to HCV, the composition and dynamics of the early antiviral response in acute infection have not been fully defined. Here, we characterize the host response to acute HCV through transcriptional profiling of peripheral blood mononuclear cells (PBMC). We performed RNA-Seq on longitudinal samples collected before, during and after acute HCV infections that resulted in spontaneous resolution or chronic infection. Our analysis provides a detailed characterization of the inflammatory and ISG signatures active in the spontaneous resolution of acute HCV, and identifies similarities with responses to flavivirus vaccines and infections.

## Results

### RNA-Seq analysis of PBMC from individuals before, during and after acute HCV infection

**Fig 1:**
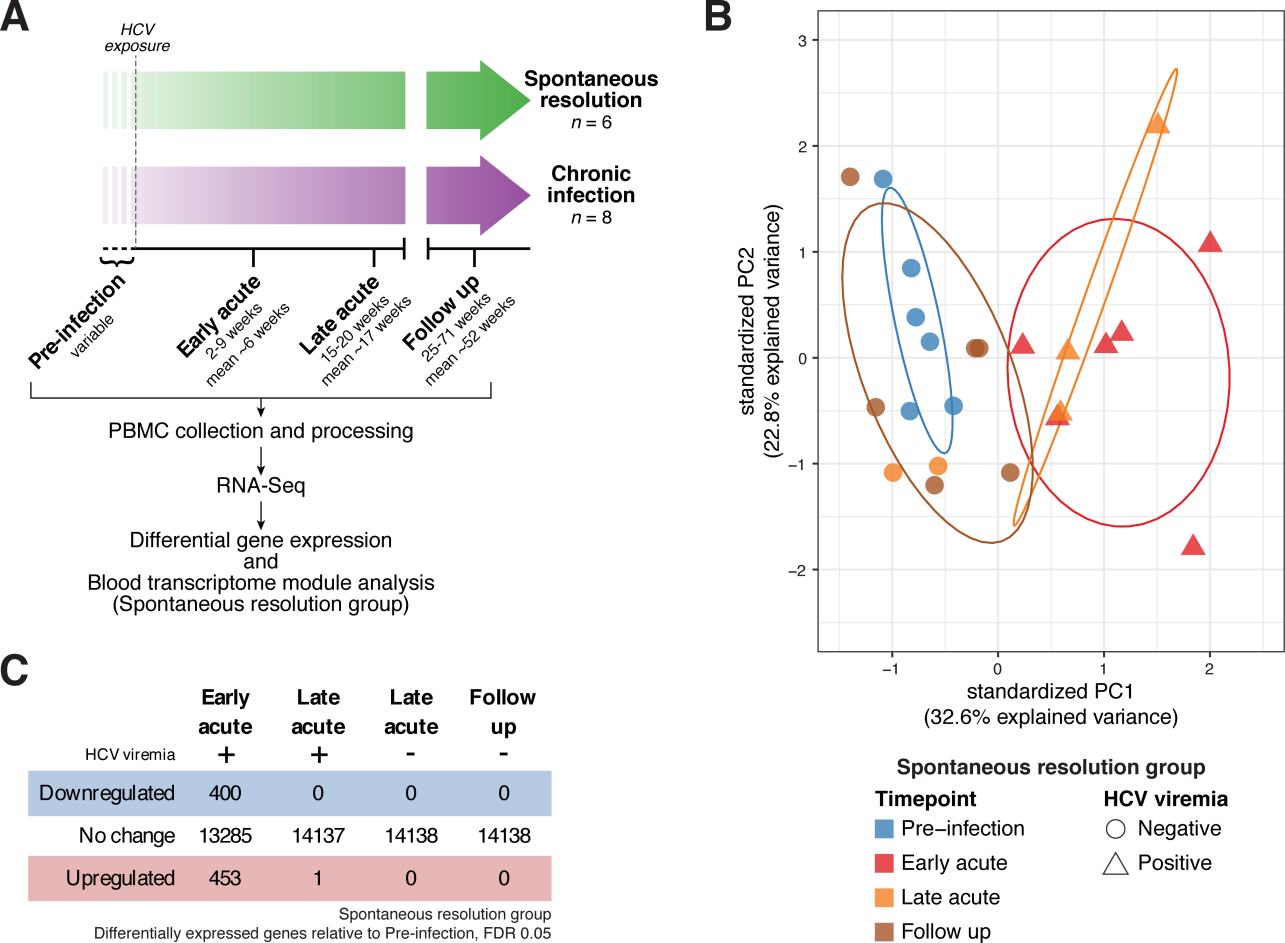
Study design and differential gene expression analysis in the Resolution group. (A) Overall study design. (B) Principal component analysis (PCA) of gene expression in the Resolution group (patient-specific gene expression variation removed as detailed in Materials and Methods). Color denotes time point, shape denotes HCV viremia status. Ellipses indicate 68% normal probability for each group (for Resolution group, Late Acute timepoint, ellipse plotted for positive HCV viremia samples only). (C) Summary of differential gene expression analysis results in the Resolution group. See also S1 and S2 Tables.

Identifying and recruiting research subjects shortly after HCV exposure is challenging due to the typically asymptomatic nature of the infection [2]. The Montreal Hepatitis C Cohort (HEPCO) recruits and follows PWID at high risk of HCV exposure and infection [21]. Longitudinal samples from this cohort provide a rare opportunity to explore the dynamics of the immune response to acute HCV. Here, we examined PBMC from 14 individuals (Tables 1 and 2) who became infected with HCV, of whom 6 spontaneously cleared the virus (Resolution group) and 8 progressed to chronic infection (Chronic group). We performed RNA-Seq on PBMC samples collected at several time points relative to HCV exposure: Pre-infection, Early acute, Late acute, and Follow up (Fig 1A) as described in Materials and methods.

**Table 1:** Patient demographics and clinical information for samples analyzed by RNA-Seq: Resolution group.

Patient Code	Sex	Age at infection	Ethnicity	rs12979860 IFNL4 genotype	HCV genotype	Testing interval (days)^a^		Estimated days post-infection	ALT (U/L)	AST (U/L)	HCV viremia
**R1**	**F**	**23**	**Indigenous**^b^	**CC**	**ND**^c^	**32**					
							*Pre-infection*	-196	29	33	-
							*Early acute*	16	199	80	+
							*Late acute*	105	42	61	+
							*Follow up*	503	31	57	-
**R2**	**M**	**44**	**Caucasian**	**CC**	**1a**	**65**					
							*Pre-infection*	-114	23	34	-
							*Early acute*	33	25	36	+
							*Follow up*	340	28	38	-
**R3**	**M**	**24**	**Caucasian**	**CC**	**3a**	**71**					
							*Pre-infection*	-35	29	37	-
							*Early acute*	48	458	334	+
							*Late acute*	126	18	26	-
							*Follow up*	392	21	40	-
**R4**	**M**	**25**	**Caucasian**	**CC**	**1**	**84**					
							*Pre-infection*	-42	24	37	-
							*Early acute*	42	2076	1335	+
							*Late acute*	138	18	30	-
							*Follow up*	409	23	37	-
**R5**	**F**	**38**	**Eastern European**	**CC**	**3a**	**84**					
							*Pre-infection*	-131	33	25	-
							*Early acute*	42	618	807	+
							*Late acute*	108	25	34	+
							*Follow up*	197	19	28	-
**R6**	**M**	**47**	**Caucasian**	**CT**	**1a**	**91**					
							*Pre-infection*	-45	20	31	-
							*Early acute*	68	449	208	+
							*Late acute*	130	37	39	+
							*Follow up*	179	23	36	-

^a^ The interval (in days) between the last negative and first positive HCV qualitative RNA test available on record. Values do not necessarily coincide with the availability of research samples or research visits

^b^ The term Indigenous respectfully refers to the First Nations, Inuit, and Métis Peoples of Canada

^c^ ND: Not Done

**Table 2:** Patient demographics and clinical information for samples analyzed by RNA-Seq: Chronic group.

Patient Code	Sex	Age at infection	Ethnicity	rs12979860 IFNL4 genotype	HCV genotype	Testing interval (days)^a^		Estimated days post-infection	ALT (U/L)	AST (U/L)	HCV viremia
**C1**	**M**	**39**	**Caucasian**	**CC**	**1a**	**76**					
							*Pre-infection*	-38	38	48	-
							*Early acute*	52	117	96	+
							*Late acute*	117	48	54	+
							*Follow up*	381	257	54	+
**C2**	**M**	**43**	**Caucasian**	**CC**	**1a**	**164**					
							*Pre-infection*	-166	33	56	-
							*Late acute*	111	53	98	+
							*Follow up*	444	34	84	+
**C3**	**M**	**33**	**Caucasian**	**CC**	**1a**	**91**					
							*Pre-infection*	-259	18	29	-
							*Early acute*	46	22	44	+
							*Late acute*	103	436	299	+
							*Follow up*	385	37	44	+
**C4**	**M**	**26**	**Caucasian**	**CC**	**3a**	**19**					
							*Pre-infection*	-342	30	30	-
							*Early acute*	37	481	294	+
							*Late acute*	110	92	46	+
							*Follow up*	381	259	175	+
**C5**	**M**	**31**	**Indigenous**^b^	**CT**	**1a**	**85**					
							*Pre-infection*	-125	35	36	-
							*Early acute*	43	287	171	+
							*Late acute*	109	181	129	+
							*Follow up*	406	87	63	+
**C6**	**M**	**27**	**Caucasian**	**CC**	**3**	**106**					
							*Pre-infection*	-53	26	25	-
							*Early acute*	53	69	58	+
							*Late acute*	114	100	96	+
							*Follow up*	374	100	50	+
**C7**	**M**	**26**	**Caucasian**	**CT**	**1a**	**162**					
							*Pre-infection*	-126	26	40	-
							*Late acute*	126	162	203	+
							*Follow up*	420	69	98	+
**C8**	**M**	**31**	**Caucasian**	**CC**	**1a**	**35**					
							*Pre-infection*	-66	18	33	-
							*Early acute*	26	110	84	+
							*Late acute*	131	54	46	+
							*Follow up*	284	38	40	+

^a^ The interval (in days) between the last negative and first positive HCV qualitative RNA test available on record. Values do not necessarily coincide with the availability of research samples or research visits

^b^ The term Indigenous respectfully refers to the First Nations, Inuit, and Métis Peoples of Canada

We first performed principal component analysis (PCA) to assess the degree to which gene expression patterns corresponded with sample group assignments. Within the Resolution group, we observed clear separation (first principal component, 32.6% explained variance, patient-specific variation removed as detailed in Materials and Methods) of the Early acute samples from Pre-infection and Follow up samples (Fig 1B, and S1A Fig). Some of the Late acute samples also separated from Pre-infection and Follow-up samples but grouped with the Early acute samples; this grouping corresponded with detectable HCV viremia. Within the Chronic group, although some general trends were apparent, PCA did not clearly separate samples according to experimental group (S1A and S1B Fig). The cause of this intersample heterogeneity in the chronic group is not clear. The Resolution and Chronic groups are similar with regard to age and ethnicity (Tables 1 and 2), and are drawn from the same populations. At this limited sample size, we suspect that confounding self-reported factors (e.g. other minor non-HCV infections, inflammatory conditions, etc.) may contribute to variation in gene expression patterns. We reasoned that, given the considerable interindividual variation apparent in human gene expression data generally, and the additional variation potentially introduced by approximate time point sampling of “real world” acute HCV infection, a statistical analysis on appropriately grouped samples would be most likely to provide high quality, generalizable results regarding the immune response to HCV. Therefore, we chose to focus the remainder of our analysis on the Resolution group, which we further partitioned based on viremia status: Early acute, Late acute (positive HCV viremia), Late acute (negative HCV viremia) and Follow up.

### Acute HCV infection elicits a dynamic host response in peripheral immune cells, which rapidly normalizes after viral clearance

We began our analysis by examining changes in individual gene expression patterns. Applying differential gene expression testing across all time point groups, we detected numerous genes whose expression values were significantly altered during acute HCV infection and resolution (*F*-test *q*-value < 0.1, S1 Table). As gene expression dynamics and PCA (Fig 1B) suggested that the most pronounced changes occurred in samples with detectable viremia, we conducted pairwise differential gene expression testing for each post-infection time point group *versus* pre-infection baseline. We detected 853 individual genes differentially expressed at the Early acute time point, with few genes meeting significance thresholds in other groups (*q*-value < 0.05, Fig 1C, S2 Table). Based on PCA (and subsequent analyses described below), the low number of significant genes detected in the Late acute, positive HCV viremia group was likely due to the small number of samples (n = 3) available for analysis. Overall, these results indicate that during acute infection, the immune response to HCV includes substantial changes to peripheral blood transcriptional signatures. These differences are most pronounced during the Early acute stage of infection, but appear to persist (at least in part) during periods of detectable viremia.

### The immune response to acute HCV infection is characterized by transcriptional changes associated with diverse immune functions, including innate antiviral defense, B cells, monocytes and inflammation

**Fig 2:**
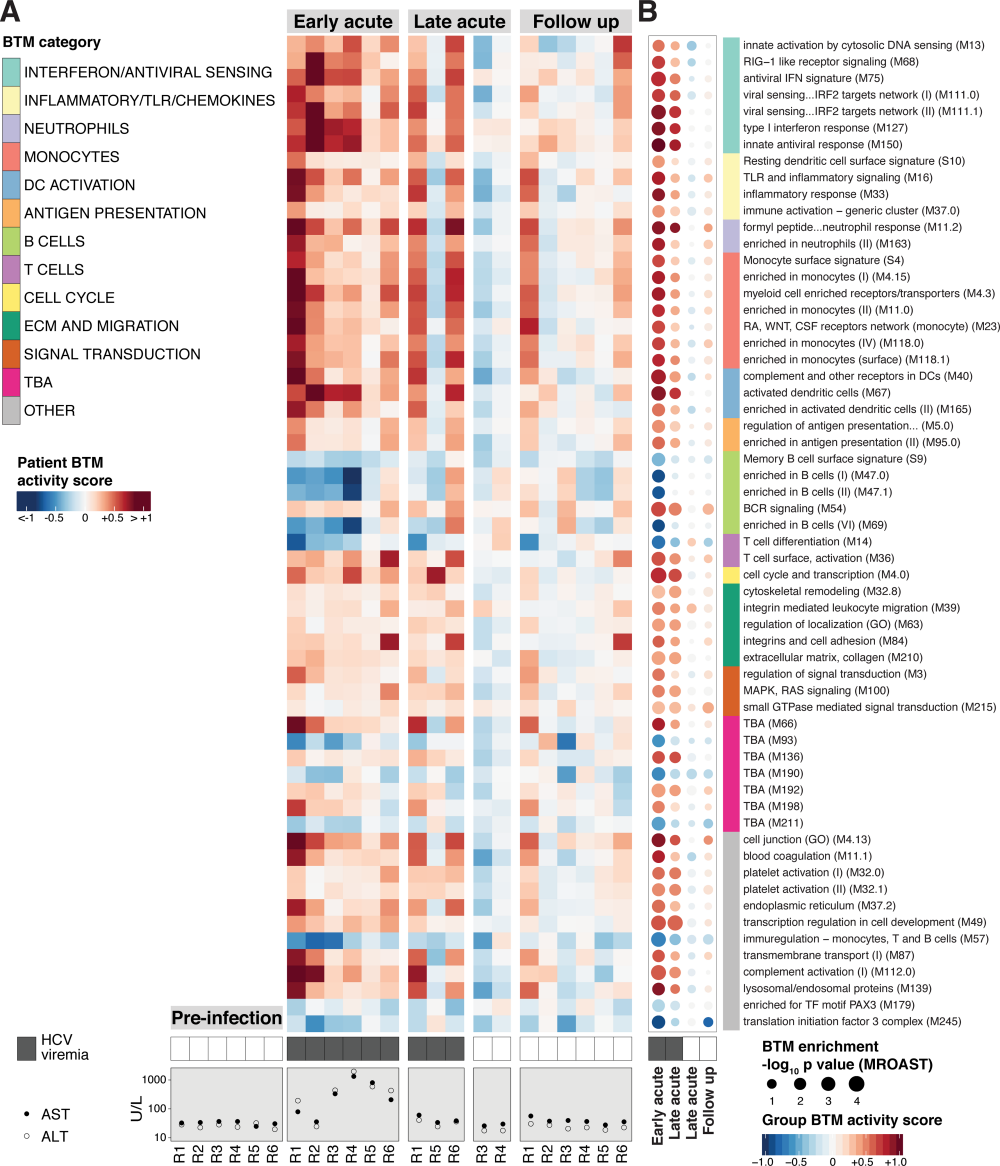
Acute HCV infection elicits a dynamic host response in peripheral immune cells. (A) Heatmap of median log_2_ fold-change sample level activity scores for the 60 BTMs differentially enriched (MROAST q-value < 0.05) at any time point (relative to Pre-Infection). Rows represent individual BTMs (category indicated on color sidebar, right), columns represent individual patient samples at indicated time points. HCV viremia (presence/absence of viral RNA by RT-PCR) and transaminase (AST, ALT) data are presented below corresponding samples. (B) “BTM dot plot” of differentially enriched BTMs by analysis group (relative to Pre-infection, any time point, MROAST q-value < 0.05). Dot color and intensity indicate direction and magnitude (group-level BTM activity score), respectively, of differential BTM enrichment. Dot size is proportional to significance (-log_10_ p-value, MROAST). BTM category indicated on color sidebar. HCV viremia status (presence/absence of viral RNA by RT-PCR) is denoted by filled/unfilled boxes at bottom of plot. See also S3 Table. BTM list order is preserved between (A) and (B).

With a goal of translating gene expression patterns to specific immune functions modulated in acute HCV, we next focused our analysis on the differential regulation of blood transcriptome modules (BTMs) [11, 22] rather than individual genes. Each BTM contains a set of genes with correlated expression patterns, annotated with associated biological functions. Using the MROAST gene set enrichment tool [23], we found 60 BTMs to be differentially enriched (*q*-value < 0.05, details in Materials and methods) during Early acute infection as compared to Pre-infection baseline (Fig 2B, S3 Table). Of note, as in the above differential gene expression analysis, changes in BTM activity were apparent in the Late acute, positive HCV viremia group but did not clear significance thresholds. At the Follow up time point, BTM activity was indistinguishable from Pre-infection levels. The activities of all differentially enriched BTMs in individual patient samples are presented in Fig 2A.

BTMs differentially enriched at the Early acute time point correspond to diverse immune functions and cell types. We further classified enriched BTMs at high level biological annotations based on categories defined by Kazmin et al [24]. Gene expression dynamics and directionality were generally similar for BTMs within the same category. Categories containing BTMs upregulated in the response to Early acute HCV include interferon/antiviral sensing, inflammatory/TLR/chemokines, monocytes, DC activation, and antigen presentation. Of note, upregulation of the “T cell surface, activation (M36)” BTM corresponded with an increased frequency of HCV-specific CD8^+^ T cells, as measured by peptide-MHC tetramer analysis (A2/NS3-1073) for HLA-A*0201^+^ patient series (n = 3, S2 Fig). Multiple downregulated BTMs were annotated in the B cell category. Taken together, these results indicate that the response to acute HCV infection involves a robust innate antiviral gene expression program in peripheral immune cells, inflammatory signals, and changes associated with innate and adaptive cell types.

### Acute HCV infection elicits a pronounced innate immune response typified by Type I IFN stimulated gene expression in peripheral immune cells

**Fig 3:**
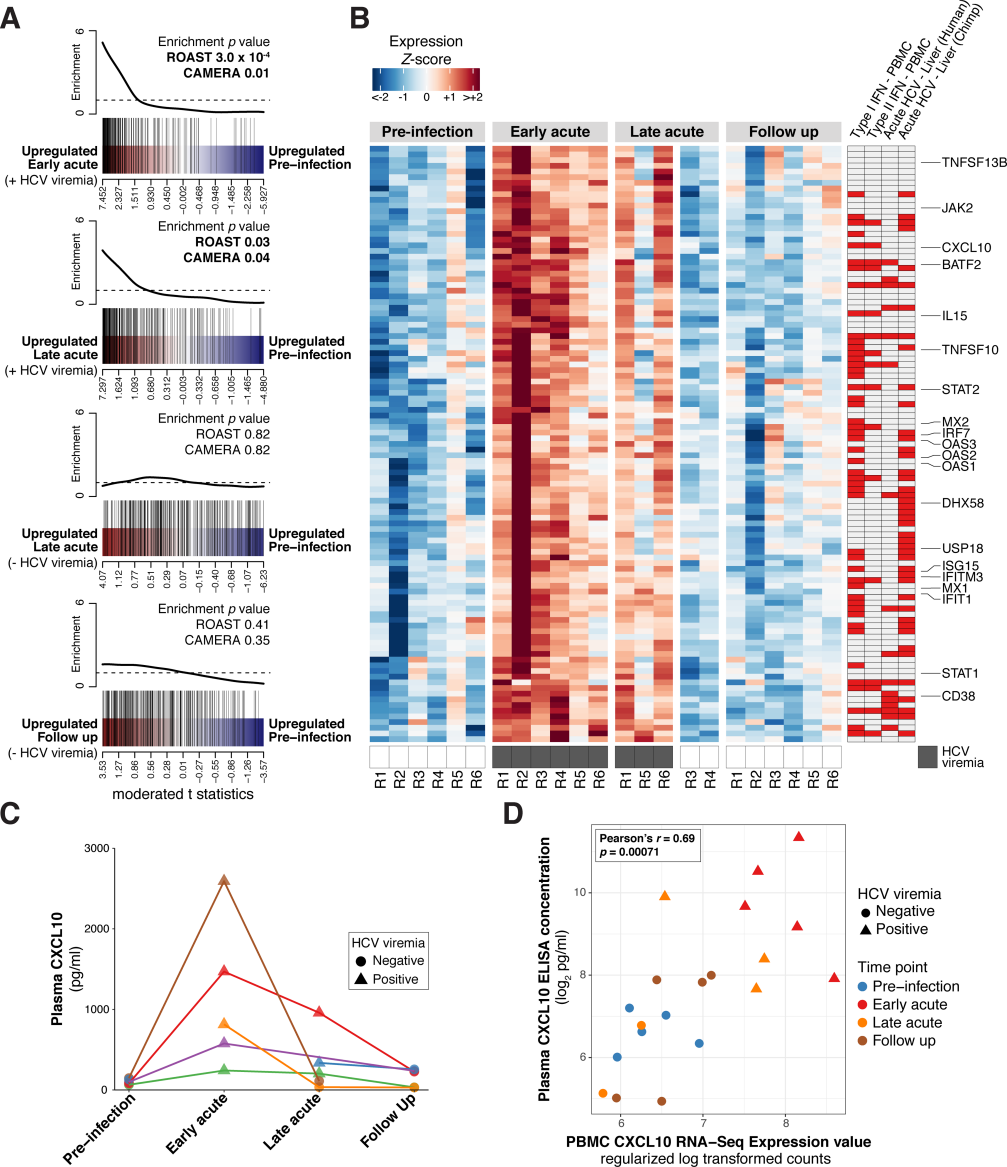
The peripheral immune response to acute HCV infection is characterized by upregulated innate antiviral/interferon signatures. (A) Barcode plots depicting differential enrichment of a PBMC ISG set for the indicated acute HCV time points relative to Pre-Infection. Red-blue color bar represents all genes ordered by differential expression statistics (moderated t statistic) for the designated contrast. PBMC ISG set member genes are highlighted by vertical black lines. Corresponding line plot displays sliding average of set enrichment. MROAST and CAMERA test p-values for PBMC ISG set enrichment are presented with each plot. (B) Heatmap displaying scaled expression values (normalized log_2_ read counts per million, scaled to z-scores by gene) for acute HCV PBMC ISG signature genes (as described in text) at Pre-Infection and Early acute time points. Select ISGs are annotated. Sidebars designate differential ISG expression in the indicated transcriptomics study (red indicates concordant upregulation): Type I IFN–PBMC and Type II IFN–PBMC [30], Acute HCV–Liver (Human) [19], Acute HCV–Liver (Chimpanzee) [25]. See also S4 Table. (C) Plasma CXCL10 concentration (pg/mL) for the indicated acute HCV time points. Each color (points/lines) denotes data from a single patient. (D) Plasma CXCL10 concentration (pg/mL) and CXCL10 RNA-Seq gene expression measures (regularized log transformed counts) from corresponding PBMC samples. Point color denotes time point analysis group, point shape denotes HCV viremia status (presence/absence of viral RNA by RT-PCR).

During acute infection, HCV triggers a potent IFN-mediated antiviral response in the liver [16, 17, 19, 25]. In our PBMC analysis, BTMs associated with the innate antiviral response were strongly upregulated during acute HCV (Fig 2, S3 Fig). At Early and Late acute time points, this upregulation corresponded with detectable HCV viremia; samples from individuals who achieved viral clearance by the Late acute time point displayed innate antiviral BTM activity similar to Pre-infection levels. Based on these results, we sought to further characterize our observations from BTM analysis using complementary reference data to describe the innate antiviral response. We tested for enrichment of a 277 gene “PBMC ISG set” empirically derived from RNA-Seq analysis of PBMC stimulated *ex vivo* with Type I IFN [26]. In agreement with BTM results, this ISG collection was significantly upregulated in the Early acute and the Late acute, positive HCV viremia time point groups, but not in the Late acute, negative HCV viremia or Follow up time point groups (Fig 3A).

We extended this analysis to define which ISGs best define the PBMC innate antiviral response to acute HCV by intersecting the PBMC ISG set with the list of differentially expressed genes at the Early acute time point (as compared to Pre-infection). The resulting list, comprised of 105 PBMC ISGs, includes ISGs previously implicated in the peripheral blood response to HCV (CXCL10) [27–29], “classical” ISGs (Mx1, OAS1, ISG15), ISG transcription factors (STAT1, STAT2, IRF7), as well as ISGs associated with immunomodulation (IL15, CD38). When intersecting this “acute HCV PBMC ISG signature” with ISG lists derived from IFN stimulated PBMC [30] or HCV liver microarray datasets (Fig 3B), we noted overlap (44/105 acute HCV PBMC ISGs) with ISG induction in acutely infected chimpanzee liver ([25], 5–11 weeks post-infection), and less overlap (16/105 acute HCV PBMC ISGs) with acutely infected human liver ([19], <6 months post-infection). This discrepancy may be due to temporal differences in liver biopsy acquisition; the human study sampled from a broader time window and described a predominantly Type II IFN signature (perhaps reflecting infiltrating adaptive immune cells later in infection). Overall, these results indicate that acute HCV infection, despite its hepatotropism, initiates a robust type I interferon response in peripheral immune cells.

To evaluate a component of this signature at the protein level, we measured levels of CXCL10 (IP-10), an important factor in the innate response to HCV [27, 31, 32], in corresponding plasma samples (available from most of the same patient-timepoint conditions examined by RNA-Seq). Similar to the patterns observed in RNA-Seq data, plasma CXCL10 levels were elevated in all samples measured at the Early acute timepoint, and later returned to baseline levels with viral clearance (Fig 3C). Furthermore, plasma CXCL10 values correlated with PBMC CXCL10 expression levels measured by RNA-Seq (Pearson’s *r* = 0.69, *p =* 0.00071) (Fig 3D). These data indicate that RNA-Seq gene expression measures in PBMC reflect the systemic protein levels of an interferon-induced chemokine during acute HCV infection.

### B cell transcriptional signatures decrease in acute HCV infection

**Fig 4:**
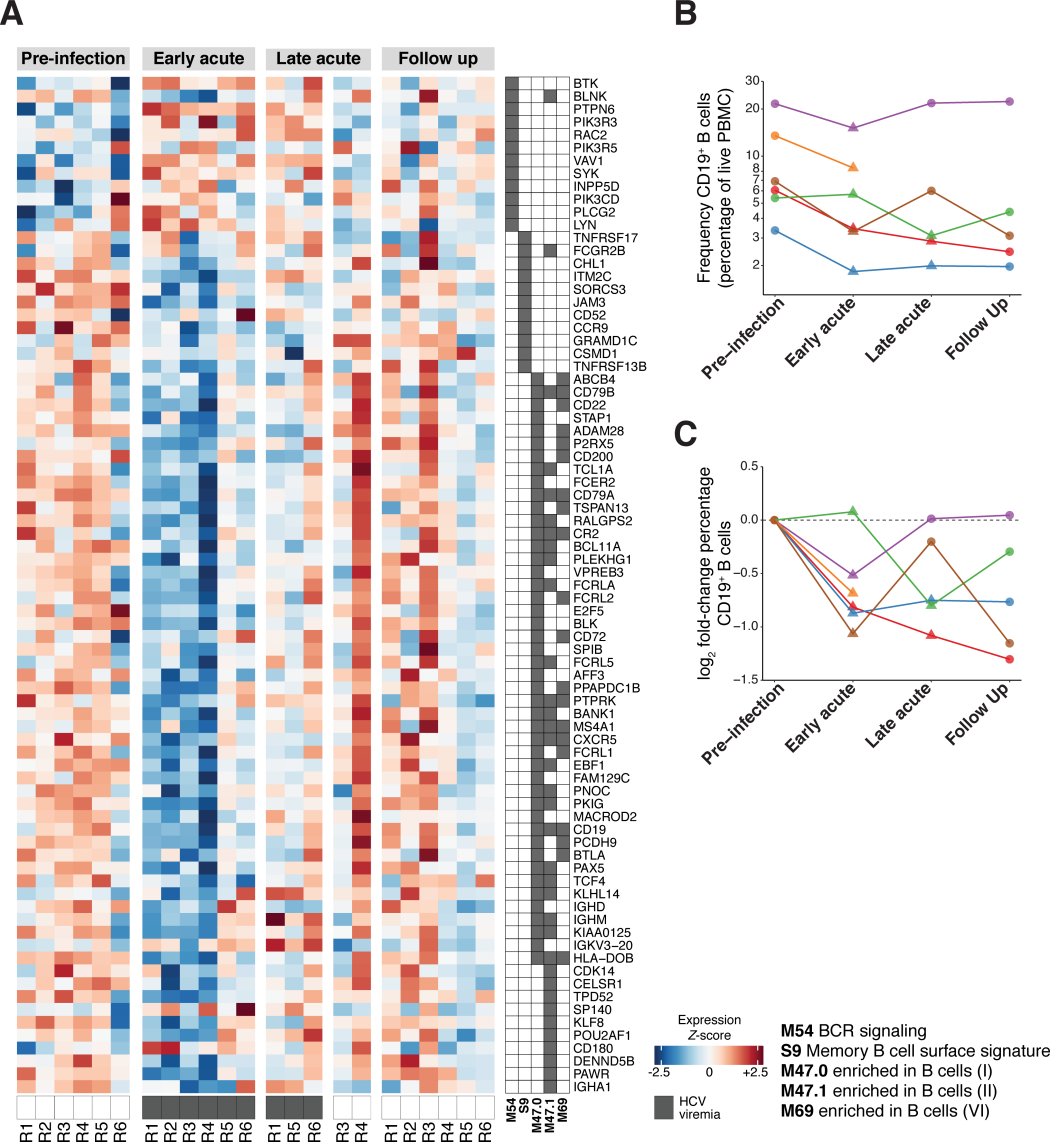
Decreased B cell transcriptional signatures in acute HCV infection. (A) Heatmap displays individual patient sample scaled expression values (normalized log_2_ read counts per million, scaled to z-scores by gene) for expressed genes composing select B cell-associated BTMs. BTM gene membership is denoted in accompanying grid annotation. Dark grey boxes along bottom of heatmap indicate detectable HCV viremia. (B) CD19^+^ B cell frequency (percentage of live PBMC as measured by flow cytometry) at indicated time points during acute HCV infection and resolution. Each color (points/lines) denotes data from a single patient. Y-axis on log_10_ scale to facilitate visualization across different patients. (C) Log_2_ fold-change (relative to corresponding patient pre-infection baseline) of CD19+ B cell frequency (percentage of live PBMC as measured by flow cytometry) at indicated time points during acute HCV infection and resolution. Each color (points/lines) denotes data from a single patient.

Several BTMs related to B cells (M47.0, M47.1, M69, S9) were found to be downregulated during Early acute HCV infection (Fig 2B). The M54 (“BCR signaling”) BTM was a notable exception and was significantly upregulated in the Early acute time point group. Most individual genes within downregulated B cell-associated BTMs demonstrated reduced expression values (Fig 4A). Rather than a decrease in gene expression output by B cells, we suspected that such a general reduction in B cell transcriptional signatures might correspond to a decrease in B cell frequency within PBMC. Therefore, we used flow cytometry to quantify the relative frequency (Fig 4B) and fold-change (Fig 4C) of CD19+ B cells in samples for which sufficient experimental material remained available. Although the limited sample numbers were insufficient to achieve statistical significance in an analysis incorporating both time point and HCV viremia status, we observed an intriguing trend of diminished B cell frequency as a fraction of total PBMC (relative to Pre-infection values, per patient) that appeared to correspond to detectable viremia at Early acute and Late acute time points (Fig 4B and 4C).

### The immune response to acute HCV shares features with the response to yellow fever vaccination

**Fig 5:**
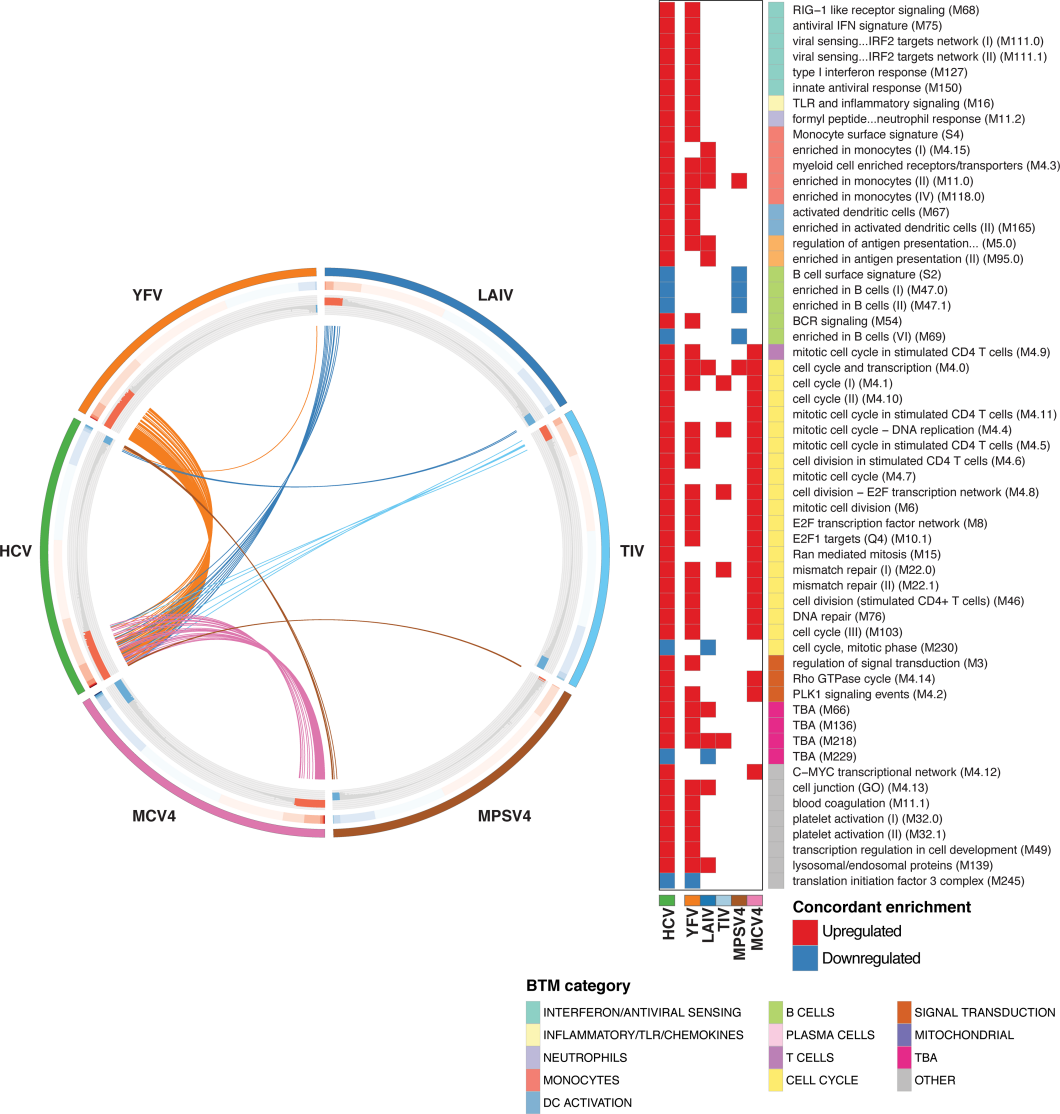
Comparison of immune responses to acute HCV and different vaccines. CIRCOS plot indicating BTM enrichment patterns shared between the Early acute HCV and indicated vaccine responses (day 7 post-vaccination versus pre-vaccination baseline). BTMs are ordered along each vaccine (or HCV) segment by GSEA Normalized enrichment score (NES) values in the corresponding dataset. Outer track color indicates HCV/vaccine dataset. Middle track heatmap plots NES values for each BTM. Inner track histogram plots -log_10_ p-values (GSEA pre-ranked) for BTM enrichment (relative to baseline); red positive bars indicate upregulation, blue negative bars indicate downregulation. For each vaccine group, each BTM demonstrating concordant activity (i.e. enriched, same directionality) with the response to Early acute HCV (Resolution group) is linked by an arc (colored by vaccine) to the HCV segment. BTMs concordant between Early acute HCV and any vaccine dataset are listed at right. Filled cells denote concordant enrichment relative to baseline (GSEA pre-ranked q-value < 0.01); red indicates upregulation, blue indicates downregulation. BTM category indicated on color sidebar.

Peripheral blood transcriptome studies have been effective in providing useful “reference” profiles of protective immune responses to different vaccines [7, 9–11, 13]. In an effort to contextualize the Acute HCV response with additional well-characterized responses to different immune challenges, we compared acute HCV PBMC transcriptional profiles to analogous, microarray studies of the PBMC response to live attenuated vaccines (yellow fever 17D, YFV; influenza, LAIV), an inactivated viral vaccine (trivalent influenza vaccine, TIV), a polysaccharide vaccine (meningococcal polysaccharide vaccine, MPSV4), and a conjugated polysaccharide vaccine (meningococcal conjugate vaccine, MCV4). After measuring BTM enrichment with GSEA (pre-ranked) [33] for each dataset (peak response vs. pre-vaccination, acute HCV infection vs. Pre-Infection), we compared responses by overlap of differentially regulated BTMs (GSEA FDR 0.01, workflow in S4 Fig). Similar to the analysis strategy described by Li et al. [11], this approach enables qualitative comparisons across methods (RNA-Seq, different microarray platforms), and modulates somewhat the statistical effects of varied sample sizes. The acute HCV response shared more significant BTMs with YFV than with any other vaccine (Fig 5). Both the acute HCV and YFV responses included interferon/antiviral sensing BTMs, inflammation-associated BTMs, and modules related to T cell proliferation.

### The immune response to acute HCV infection resembles the brief, early response to acute DENV infection, but is sustained over many weeks

**Fig 6:**
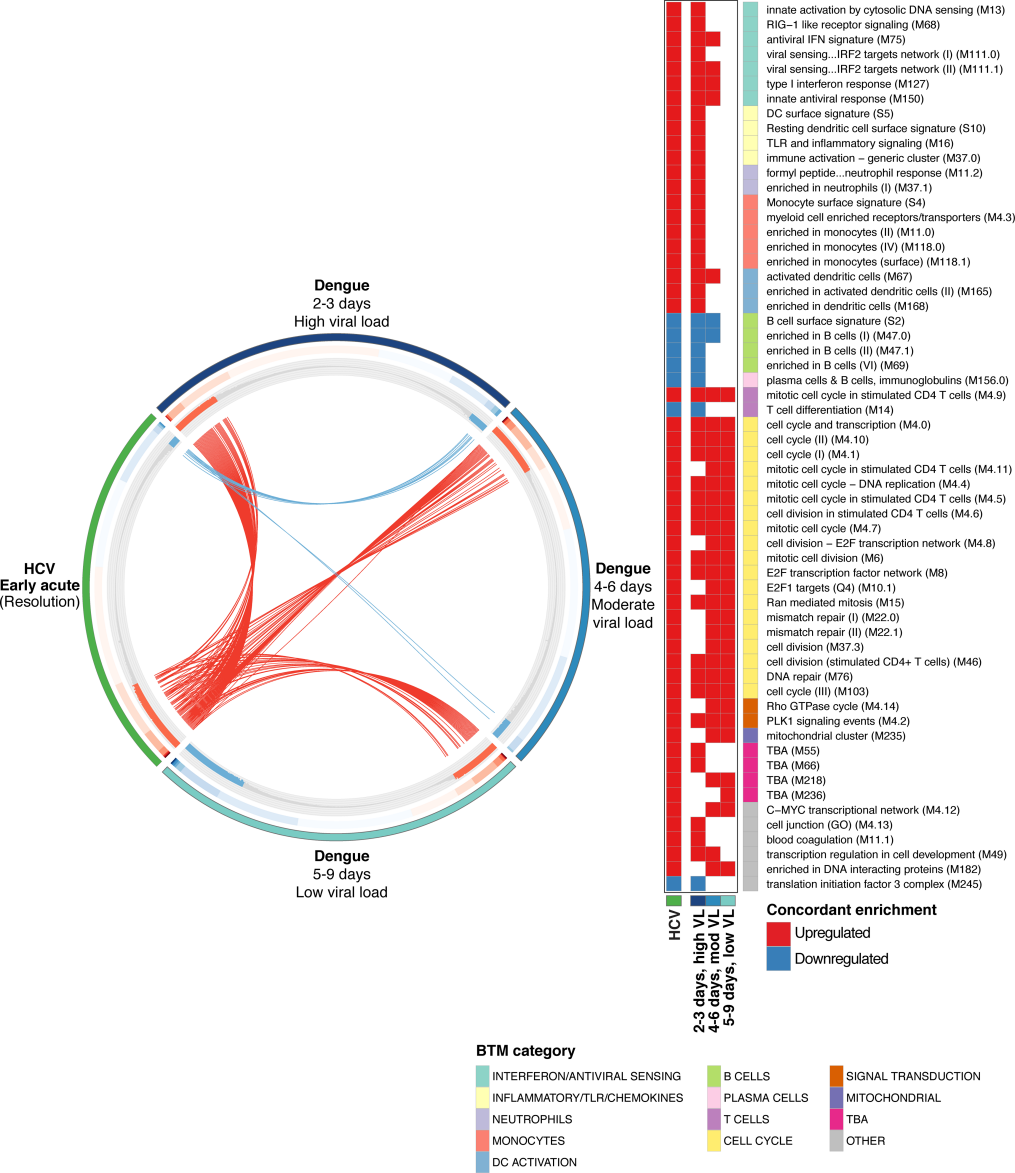
Comparison analysis of immune response to acute HCV and acute DENV infection. CIRCOS plot indicating BTM enrichment patterns shared between the Early acute HCV and acute DENV responses (DENV subgroups as defined in the main text). BTMs are ordered along each DENV subgroup (or HCV) segment by GSEA NES values in the corresponding dataset. Outer track color indicates HCV/DENV dataset. Middle track heatmap plots NES values for each BTM. Inner track histogram plots -log_10_ p-values (GSEA pre-ranked) for BTM enrichment (relative to baseline); red positive bars indicate upregulation, blue negative bars indicate downregulation. For each DENV subgroup, each BTM demonstrating concordant activity (i.e. enriched, same directionality) with the response to acute HCV is linked by an arc to the HCV segment. Each arc is colored by directionality of concordant enrichment (red indicates upregulation, blue indicates downregulation). BTMs concordant between acute HCV and any DENV subgroup dataset are listed at right. Filled cells denote concordant enrichment relative to baseline (Both acute HCV and indicated DENV subgroup datasets GSEA pre-ranked q-value < 0.01); red indicates upregulation, blue indicates downregulation. BTM category indicated on color sidebar.

Although the above vaccine comparisons provide informative functional context regarding the nature of the acute HCV response as compared to defined immune challenges, vaccines, by definition, are not full potency infections. Therefore, in order to evaluate our HCV results in relation to a *bona fide* viral infection, we compared the acute HCV response (RNA-Seq data described here) to the response mounted against acute DENV infection (publicly available whole blood microarray data) [15]. First, we partitioned the DENV dataset into three groups based on distinct gene expression profiles (PCA analysis, S5 Fig); similar groupings were observed by Kwissa *et al* in their original analysis. These groups correspond to viral load and time post-symptom onset: High viral load (2–3 days), Moderate viral load (4–6 days), and Low viral load (5–9 days). Next, we measured BTM enrichment (acute infection groups *versus* matched convalescence “baselines”) and assessed which BTMs were similarly regulated in acute resolving HCV and acute resolving DENV infection. We observed considerable overlap in the BTM response to acute HCV and acute DENV, which included many of the modules also identified in the YFV comparison (Fig 6). Concordant enrichment of BTMs was most apparent in the DENV High viral load (2–3 days) condition, with increased activity of innate antiviral BTMs, inflammation BTMs and T cell proliferation BTMs, and decreased activity of B cell BTMs. Although upregulation of BTMs related to T cell proliferation seems to persist through lower viral load/later DENV time points, many BTMs associated with the innate response (inflammatory/TLR/chemokines, monocytes, a subset of interferon/antiviral sensing BTMs) do not appear to be differentially enriched beyond the High viral load (2–3 days) group. This analysis suggests that during acute DENV infection, the early host response is characterized by inflammatory signals, B cell changes, and an innate antiviral signature, which is diminished as the response incorporates adaptive immune functions (i.e. T cell proliferation). Although many of the same BTMs are involved, this temporal pattern is in sharp contrast to the timeline of the acute HCV response. At the Early acute stage (approximately 6 weeks post-infection), the HCV response appears similar to the apparently short-lived (days) initial response to acute DENV infection.

## Discussion

Here we present a detailed transcriptomic characterization of early events in the human immune response to acute HCV infection in individuals that progress to spontaneous resolution. We detected wide-ranging changes in PBMC gene expression patterns, including those consistent with pronounced innate antiviral programs and inflammatory mediators. The patterns observed in the PBMC response to acute HCV shared many features with effective immune responses to YFV and acute DENV infection. To our knowledge, this study represents the first longitudinal transcriptomic investigation of the PBMC response to acute HCV in humans.

Studying the early immune response to HCV in humans is challenging, as infected individuals are not usually recognized until they progress to chronic infection. Even if research subjects are identified during acute stages, obtaining pre-infection samples for comparison is often impossible. We addressed these difficulties in a rare longitudinal study of HCV-naïve PWID [21, 34], through which we obtained acute infection and corresponding pre-infection baseline samples. Given this “real world” setting, we observed considerable variability in datasets from different patients. Differences in gene expression patterns could be due to complex factors associated with the PWID population enrolled in this study, including drug use, unstable socioeconomic conditions and exposures to additional (non-HCV) infections. Furthermore, the time of HCV infection is necessarily estimated and the samples grouped for each time point are not perfectly synchronized. Problems in assigning datasets to distinct analysis groups were particularly pronounced in samples from individuals who eventually progressed to chronic infection. Although reasons for greater consistency of expression patterns in the Resolution group remain unclear, much of the variation in the Chronic group samples appears unrelated to HCV infection timepoint (S1 Fig). We do not believe this heterogeneity is related to infection outcome. These issues might be overcome with a larger sample size, which was unfortunately not possible in the present study.

This study’s original goal was to identify disparities in the initial response to infection that contribute to the differential outcomes of spontaneous infection versus chronicity. Indeed, recently reported analyses of HCV-specific T cells suggest that differences in metabolic networks engaged early in infection contribute to viral clearance [18]. In our datasets, the heterogeneous gene expression patterns measured in the Chronic group precluded a direct comparison to spontaneous resolution at this sample size. However, this longitudinal study did enable a detailed characterization of the peripheral immune response to acute HCV infection that results in spontaneous resolution. Within the Resolution group, we structured our analysis to evaluate changes relative to patient-specific Pre-infection samples and we observed several consistent and concordant transcriptomic patterns across most subjects analyzed. With the range of estimated infection dates, these results suggest that certain immune functions are active to some extent for at least several weeks during acute HCV.

Our results define a pronounced innate antiviral gene expression program active in PBMC during the Early acute stage of HCV infection. This response persisted through the Late acute time point in individuals with detectable HCV viremia. These observations are consistent with transcriptomic data from the chimpanzee model demonstrating rapid induction of ISGs in the liver during early acute HCV [16, 17]. Furthermore, we observed overlap in the PBMC ISG signature identified here and ISG expression patterns in acute HCV liver biopsy samples from infected patients and chimpanzees [19, 25]. This suggests that the innate antiviral response against acute HCV is not restricted to the liver and that the peripheral blood response corresponds to some extent with that at the site of infection. This response returned to baseline levels following viral clearance, suggesting a dependence on viral RNA.

We also observed a decrease in gene signatures associated with B cells. Such changes could reflect a decrease in B cell transcriptional activity, a decrease in the relative frequency of B cells, or both. Based in part on trends observed in flow cytometry analysis of a limited number of samples, we speculate that these patterns result from a diminished fraction of B cells within PBMC during acute HCV viremia. Although it is possible that this pattern could simply be a consequence of a corresponding relative increase in another cell type (e.g. monocytes, as suggested by BTM enrichment), we did not detect significant decreases in BTMs associated with other cell types (e.g. T cells, NK cells). As both RNA-Seq (as applied here to bulk PBMC samples) and flow cytometry are relative quantification methods, we cannot ascertain if these changes correspond to a decrease in the absolute frequency of B cells in peripheral blood. However, several recent studies have described mechanisms of virus- and IFN- mediated B cell dysregulation [35–37]. Future studies including sample collection focused on absolute immune cell quantification will be required to further explore this observation in the context of acute HCV infection.

Our comparative analyses revealed notable similarities between host responses to acute HCV and to effective vaccines. More specifically, we observed many shared features with the response to YFV, a live attenuated flavivirus that may be thought of as approximating an acute viral infection. As yellow fever virus is a related hepatotropic flavivirus, these similarities are not entirely surprising. In addition, the immune response during Early acute HCV infection shared many features with the immune response to acute DENV infection. Although challenging to make a formal comparison due to discrepancies in study design and timing, these qualitative results suggest that at least at some point during acute HCV, a response similar to that elicited by DENV is induced, likely reflecting the core innate antiviral programs activated in response to RNA virus infection. However, in acute HCV, ISG expression remains elevated long after initial infection and is maintained for many weeks of detectable viremia. This pattern is consistent with models in which adaptive immunity to HCV is delayed as compared to flavivirus infections, despite apparently similar robust IFN responses [16, 38, 39]. Even in the case of eventual spontaneous resolution, HCV outpaces these innate antiviral effectors, resulting in prolonged high-level viremia [38, 39]. Eventually, HCV-specific CD4 and CD8 T cells arise to eliminate the virus, after which innate antiviral signatures rapidly normalize. Failure to prime effective adaptive immunity results in chronic infection that is associated with persistent ISG expression (reviewed in [3, 4]).

This longitudinal study provides an initial assessment of the peripheral immune response to acute HCV at a systems level. Follow-up studies with a larger cohort and sufficient sample availability for complementary experimental methods (e.g. comprehensive flow cytometry analysis) will be required to validate these findings beyond the patients described here and determine the impact of these dynamically regulated immune processes on the differential outcome of acute HCV infection.

## Materials and methods

### Ethics statement

This study was approved by the ethics committee of the Centre Hospitalier de l'Université de Montréal (CHUM) (Protocol SL05.014). All research was conducted according to the principles expressed in the Declaration of Helsinki. All subjects provided written informed consent.

### Patients and samples

Subjects with acute HCV were recruited among high-risk PWID participating in the HEPCO cohort as previously described [21, 34]. Estimated date of infection (EDI) was calculated as the median date between the last HCV negative and the first HCV positive test. Spontaneous viral resolution (*n*  =  6) or chronic infection (*n*  =  8) was defined as the absence or presence of HCV RNA, respectively, at 6 months post EDI (Cobas Ampliprep/Cobas TaqMan HCV Qualitative Test, version 2.0; Limit of detection: 15 IU/ml)). PBMC samples from four time points were examined: i) Pre-infection baseline (Variable); ii) Early acute (2–9 weeks, mean 6 weeks); iii) Late acute (15–20 weeks, mean 17 weeks); and iv) Follow up (25–71 weeks, mean 52 weeks). Subjects’ clinical characteristics, demographics, testing intervals and actual times post EDI for each sample are presented in Tables 1 and 2.

### RNA-Seq library preparation and data processing

Cryopreserved PBMC samples were thawed, viability was assessed using trypan blue (>90% for all samples) and immediately processed for RNA extraction (approximately 7x10^6^ PBMC per sample, range: 4 x 10^6^–11 x 10^6^) with the Qiagen RNEasy Mini kit. RNA quality was assessed by Agilent Bioanalyzer 2100; all samples exhibited RNA integrity numbers (RIN) greater than 8. RNA-Seq libraries were prepared with the Illumina TruSeq RNA Library Preparation Kit v2. Libraries were sequenced in multiplex on the Illumina HiSeq 2500 platform in 100 nucleotide, single-end read configuration (range 3 x 10^7^–7 x 10^7^ total reads per library). Samples from the same patient series were always sequenced in the same multiplex pool to minimize batch effects.

Reads were mapped to the human genome reference (hg19) using the Tophat (v2.0.8b) alignment tool [40]. Read counts per gene were quantified against Ensembl (v66) transcript reference annotations (appended with gene annotation for *IFNL4*) using HTSeq-count (v0.5.4p3) [41].

### Acute HCV gene expression and BTM analysis

Analysis was conducted within the R statistical framework. For principal component analysis, read counts were normalized and variance stabilized by regularized log transformation (rlog() function, DESeq2 package v1.18.1). Patient-specific gene expression variation was corrected using the removeBatchEffect() function in the limma package (v3.26.9), specifying time point and viremia status for preservation. For independent Spontaneous Resolution (Fig 1B) and Chronic infection (S1B Fig) PCAs, analysis included the top 500 most variable genes across samples from the indicated infection group. For joint Chronic Infection and Spontaneous Resolution PCA (S1A Fig), PCA was performed on a single gene list (top 1000 most variable genes across all samples), and Chronic and Resolution groups were plotted separately to facilitate visualization.

In order to incorporate the multiple covariates apparent in the experimental design, differential expression and BTM enrichment analyses were conducted with the voom-limma analysis workflow (v3.26.9) [42]. Prior to differential expression analysis, a filter was applied to remove genes with low expression values (genes with greater than one read count per million (cpm) in at least four samples were designated as “expressed”); 14,138 genes passed filter and were included in subsequent analyses. RNA-Seq read counts were scaled and normalized by the trimmed mean of M values (TMM) method (implemented in the edgeR package [43, 44]) and log_2_ transformed using voom [45]. All differential gene and gene set analyses were based on a linear model specifying covariates for patient, and a categorical joint factor incorporating time point and viremia status (Pre-infection, Early acute [positive viremia], Late acute [positive viremia], Late acute [negative viremia], Follow up). Differential gene and gene set analyses were adjusted for multiple testing by the method of Benjamini and Hochberg [46].

BTM gene memberships and annotations were obtained from Li et al [11], and enrichment tests were performed with MROAST [23] and the above described linear model. The MROAST tool was selected due to its capacity for complex experimental designs and voom-generated gene weights. Group level BTM activity scores were derived from the proportion of genes in a given BTM contributing to significance (i.e. genes in BTM with |z| > √2) as reported by MROAST, with sign indicating direction of enrichment relative to Pre-infection baseline. For visualizing BTM changes per individual patients (Fig 2A), sample level fold-change activity scores were calculated as the median log_2_ fold-changes (sample time point vs. Pre-infection, per patient) of BTM member genes for each module.

### Acute HCV PBMC ISG signature

PBMC ISG set [26] enrichment testing was performed with ROAST [23] and CAMERA [47], using the above described linear model. Genes within the PBMC ISG set found to be differentially expressed (*q* value < 0.05, S4 Table) at the Early acute time point relative to Pre-infection baseline were selected as an “Acute HCV PBMC ISG signature.” The Acute HCV PBMC ISG signature gene list was intersected with lists of differentially expressed genes in different biological contexts as reported by corresponding publications: Acute HCV, Human Liver [19]; Acute HCV, Chimpanzee liver [25]; Type I IFN and Type II IFN, Human PBMC ex vivo [30].

### BTM enrichment comparison: Acute HCV infection and vaccine signatures

Microarray data for PBMC responses to the following vaccines were obtained from the GEO database: YFV (GSE13485) [9], LAIV (GSE29615) and TIV (GSE29617) [48], MPSV4 (GSE52245) and MCV4 (GSE52245) [11]. In an effort to minimize the impact of different study designs, transcriptome platforms (RNA-Seq, different microarrays) and sample sizes, the GSEA (pre-ranked) approach was used to evaluate BTM activity in each individual dataset as follows (S4 Fig). For each vaccine dataset, differential gene expression analysis for peak response (day 7) versus pre-vaccination baseline (day 0) was performed with limma in parallel analysis workflows. Only those genes represented in all datasets were maintained for subsequent analysis. For each dataset, genes were ranked by moderated *t*-statistics, and input to the GSEA (pre-ranked) module on GenePattern (http://genepattern.broadinstitute.org). Comparative profiles of BTMs enriched in both Acute HCV and vaccine datasets (CIRCOS plots) were defined as the intersect of modules enriched at GSEA *q* value < 0.01 (concordant direction of up/down regulation) for each dataset (for Acute HCV, time point *vs*. Pre-Infection; for vaccines, peak response versus baseline).

### BTM enrichment comparison: Acute HCV infection and acute DENV infection

Microarray data on the peripheral whole blood response to acute DENV infection [15] were obtained from GEO (GSE51808). Samples without paired convalescent controls were not included in analysis. Principal component analysis was used to identify distinct infection groups based on gene expression patterns: High viral load (2–3 days), Moderate viral load (4–6 days), and Low viral load (5–9 days). Differential gene expression analysis (acute DENV infection versus paired convalescent “baseline” controls) for each infection group was performed with limma. BTM profiles for each Acute HCV and DENV group were generated using the GSEA (pre-ranked) approach as described for vaccine comparisons.

### Flow cytometry

Cryopreserved PBMCs were thawed and analyzed using different panels for T cells and B cells phenotyping against the following markers: CD3 (clone UCHT1), CD4 (clone RPA-T4), CD8 (clone SK1), CD10 (clone HI10a), CD19 (clone AJ25C1), CD20 (clone 2H7), CD21 (clone B-ly4), CD38 (clone HB7), CD56 (clone NCAM 16.2), IgM (clone G20-127), HLA-DR (G46-6), all from BD Bioscience (San Diego, CA); CD1 (clone L161), CD27 (Clone O323) both from Thermo-Fisher (Waltham, MA); CD22 (Clone HIB22), IgG (clone M1310G05) both from BioLegend (San Diego, CA). HLA-A2/NS3-1073 tetramers (HLA-A2 restricted HCV-NS3 peptide aa 1073–1081 (CINGVCWTV)) were obtained from the NIH Tetramer Core facility (Emory University, Atlanta, GA. Multiparameter flow cytometry was performed at the flow cytometry core of the CRCHUM using a BD LSRII instrument equipped with violet (405 nm), blue (488 nm), yellow-green (561 nm) and red (633 nm) lasers and FACSDiva version 8.0.1 (BD Biosciences). FCS data files were analyzed using FlowJo version 10.0.8 for Mac (Tree Star, Ashland, OR). Fluorescence minus one controls were used to set the analysis gates.

### CXCL10 ELISA

CXCL10 levels in plasma were quantified using the human CXCL10/IP-10 Quantikine ELISA Kit (R&D Systems Inc, Minneapolis, MN) according to the manufacturer’s protocol.

## Supporting information

S1 Fig Principal component analysis (PCA) of Resolution and Chronic group samples. (A) Joint PCA for Resolution and Chronic group samples. PCA was conducted on variance stabilized gene-level read counts, with correction for patient-specific gene expression variation as detailed in *Materials and Methods*. PCA included a single gene list (top 1000 most variable genes across all samples), and Chronic and Resolution groups were plotted separately to facilitate visualization. Ellipses indicate 68% normal probability for each group (for Resolution group, Late Acute timepoint, ellipse plotted for positive HCV viremia samples only).
(B) PCA for Chronic group samples. PCA was conducted on variance stabilized gene-level read counts, with correction for patient-specific gene expression variation as detailed in *Materials and Methods*. PCA was performed on the top 500 most variable genes across all Chronic group samples. Ellipses indicate 68% normal probability for each group.
(PDF)

S2 Fig Frequency of HCV-specific CD8+ T cells during Acute HCV infection. (A) Example of HCV-tetramer (HLA-A2/NS3-1073) labeling as measured by flow cytometry (Patient R1). Values indicate percentage of tetramer^+^ events in Live, CD3^+^CD8^+^ gate.
(B) Line plot displaying HCV-specific T cell frequencies as measured by flow cytometry for n = 3 patients. Values indicate percentage of tetramer+ (HLA-A2/NS3-1073) events in Live, CD3^+^CD8^+^ gate.
(PDF)

S3 Fig PBMC interferon/innate antiviral response to acute HCV infection. Heatmap displays individual sample scaled expression values (normalized log_2_ read counts per million, corrected for patient-specific variation, scaled to z-scores by gene) for expressed genes composing enriched BTMs in the INTERFERON/ANTIVIRAL SENSING category. BTM gene membership is denoted in accompanying grid annotation. Dark grey boxes along bottom of heatmap indicate detectable HCV viremia.
(PDF)

S4 Fig Schematic workflow for Comparative BTM enrichment analysis (Acute HCV infection and vaccine signatures). Full details in Materials and methods section.
(PDF)

S5 Fig Principal component analysis (PCA) to determine sample groupings for acute DENV infection microarray datasets. PCA was conducted on all samples with infection and paired convalescence controls from GSE51808 [15]. After correction for sex-specific gene expression variation by the RemoveBatchEffect() function in limma, PCA was performed on the top 1000 most variable genes across all included samples. After assigning sample groupings based on initial PCA, plot colors and ellipses were included to facilitate visualization of group assignments. Ellipses indicate 68% normal probability for each group.
(PDF)

S1 Table Differentially expressed genes across all Resolution timepoint group conditions (F test, FDR 0.1). Columns labeled with timepoint group denote log_2_ fold-change in gene expression for the indicated condition relative to Pre-Infection baseline.
(TXT)

S2 Table Differentially expressed genes, Resolution group, Early acute versus Pre-Infection baseline (FDR 0.05). (TXT)

S3 Table Differentially enriched BTMs, Resolution group, Early acute versus Pre-infection baseline (MROAST FDR 0.05). (TXT)

S4 Table Acute HCV PBMC ISG signature. Genes within the PBMC ISG set found to be differentially expressed (Resolution group, Early acute versus Pre-infection baseline, *q* value < 0.05) with associated statistics.
(TXT)
