# Supplementary figures and images for: Longitudinal transcriptomic characterization of the immune response to acute hepatitis C virus infection in patients with spontaneous viral clearance
Source: PLoS Pathog. 2018 Sep 17;14(9):e1007290. doi: 10.1371/journal.ppat.1007290 (PMC6160227; doi:10.1371/journal.ppat.1007290)

A

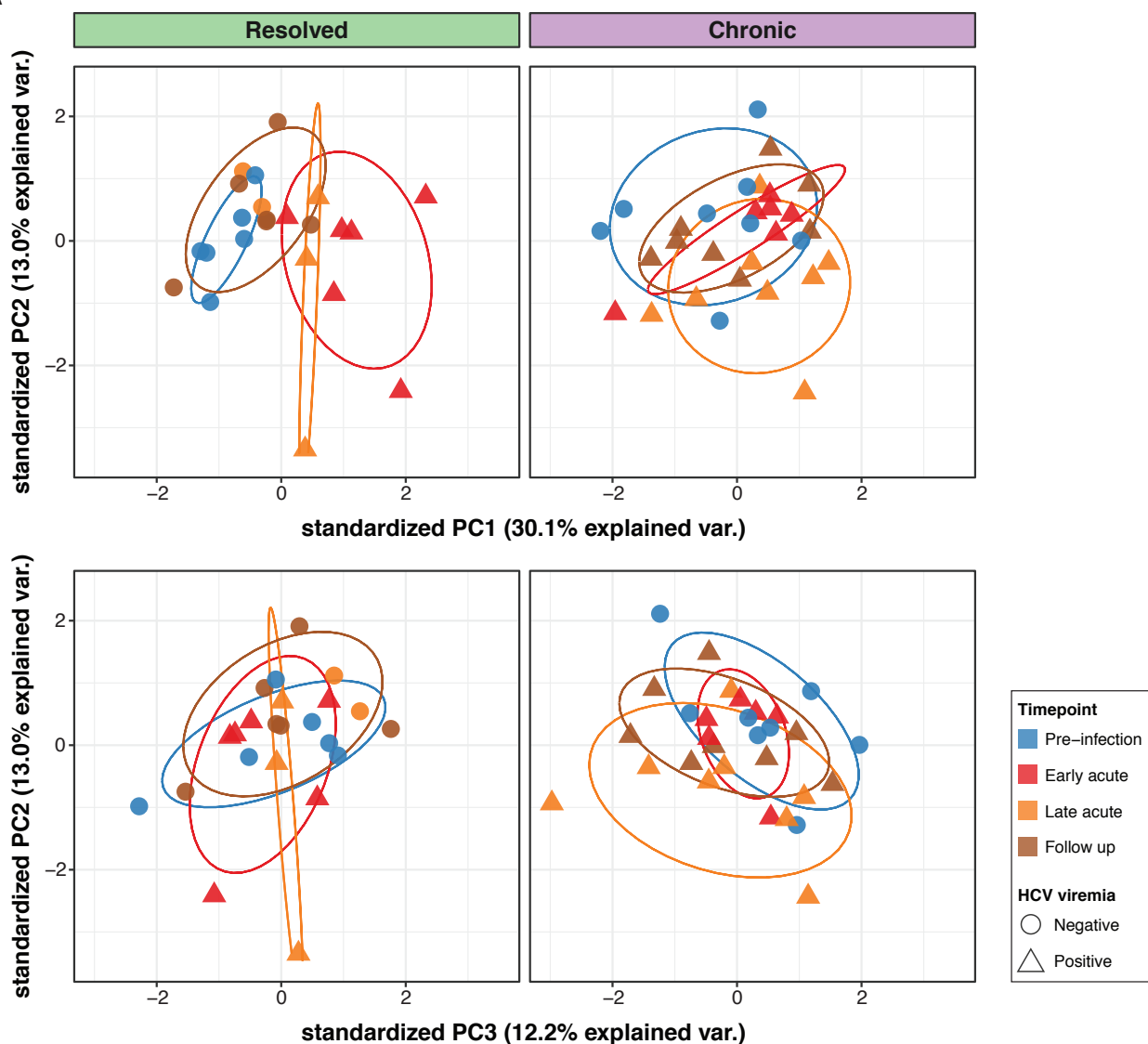

B

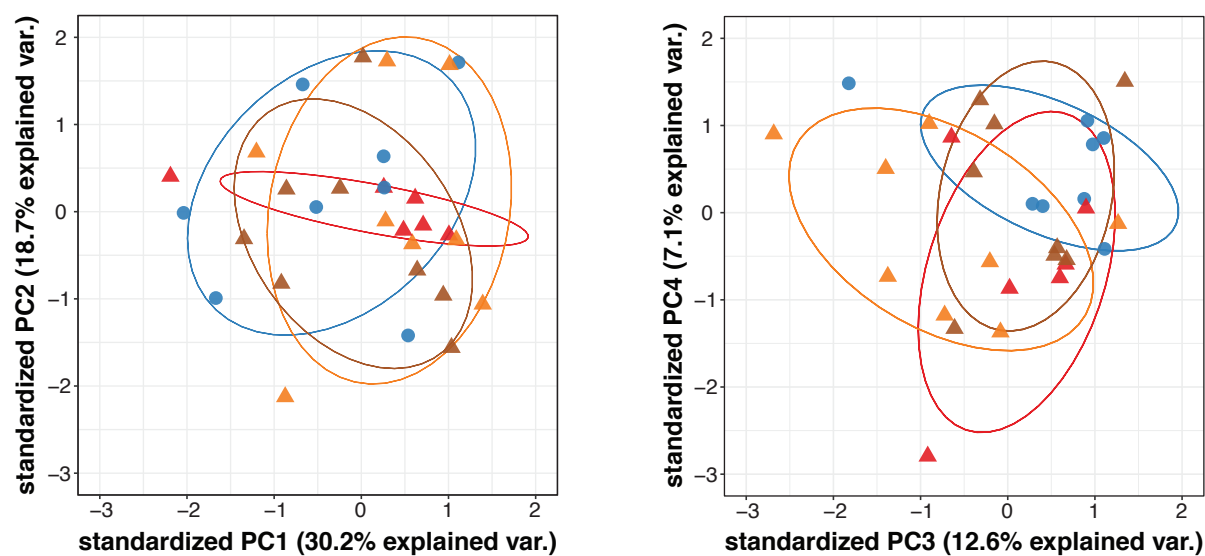

Supplement: S1 Fig — (A) Joint PCA for Resolution and Chronic group samples. PCA was conducted on variance stabilized gene-level read counts, with correction for patient-specific gene expression variation as detailed in Materials and Methods. PCA included a single gene list (top 1000 most variable genes across all samples), and Chronic and Resolution groups were plotted separately to facilitate visualization. Ellipses indicate 68% normal probability for each group (for Resolution group, Late Acute timepoint, ellipse plotted for positive HCV viremia samples only). (B) PCA for Chronic group samples. PCA was conducted on variance stabilized gene-level read counts, with correction for patient-specific gene expression variation as detailed in Materials and Methods. PCA was performed on the top 500 most variable genes across all Chronic group samples. Ellipses indicate 68% normal probability for each group. (PDF) [file ppat.1007290.s001.pdf]

**A**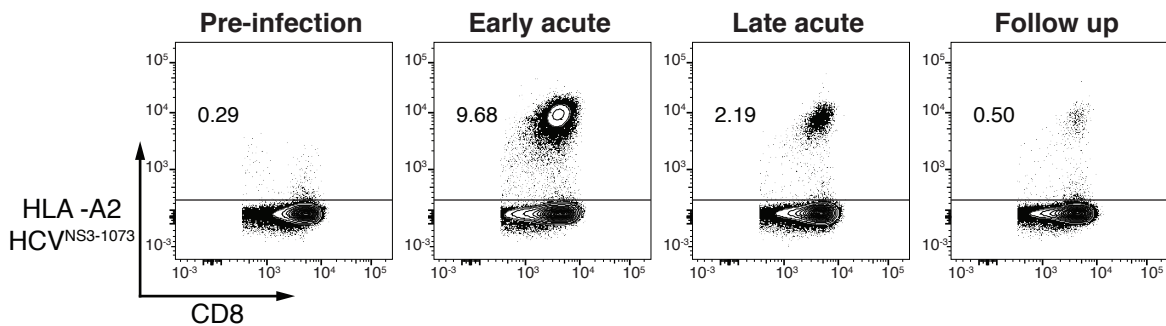**B**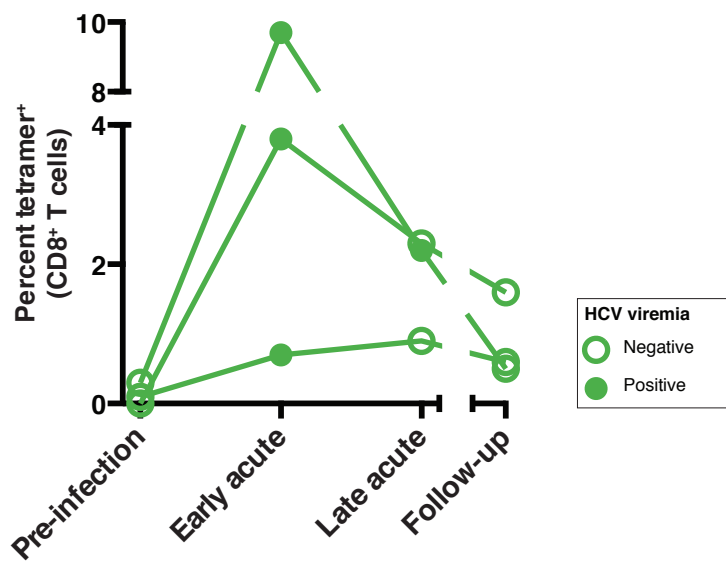

Supplement: S2 Fig — (A) Example of HCV-tetramer (HLA-A2/NS3-1073) labeling as measured by flow cytometry (Patient R1). Values indicate percentage of tetramer+ events in Live, CD3+CD8+ gate. (B) Line plot displaying HCV-specific T cell frequencies as measured by flow cytometry for n = 3 patients. Values indicate percentage of tetramer+ (HLA-A2/NS3-1073) events in Live, CD3+CD8+ gate. (PDF) [file ppat.1007290.s002.pdf]

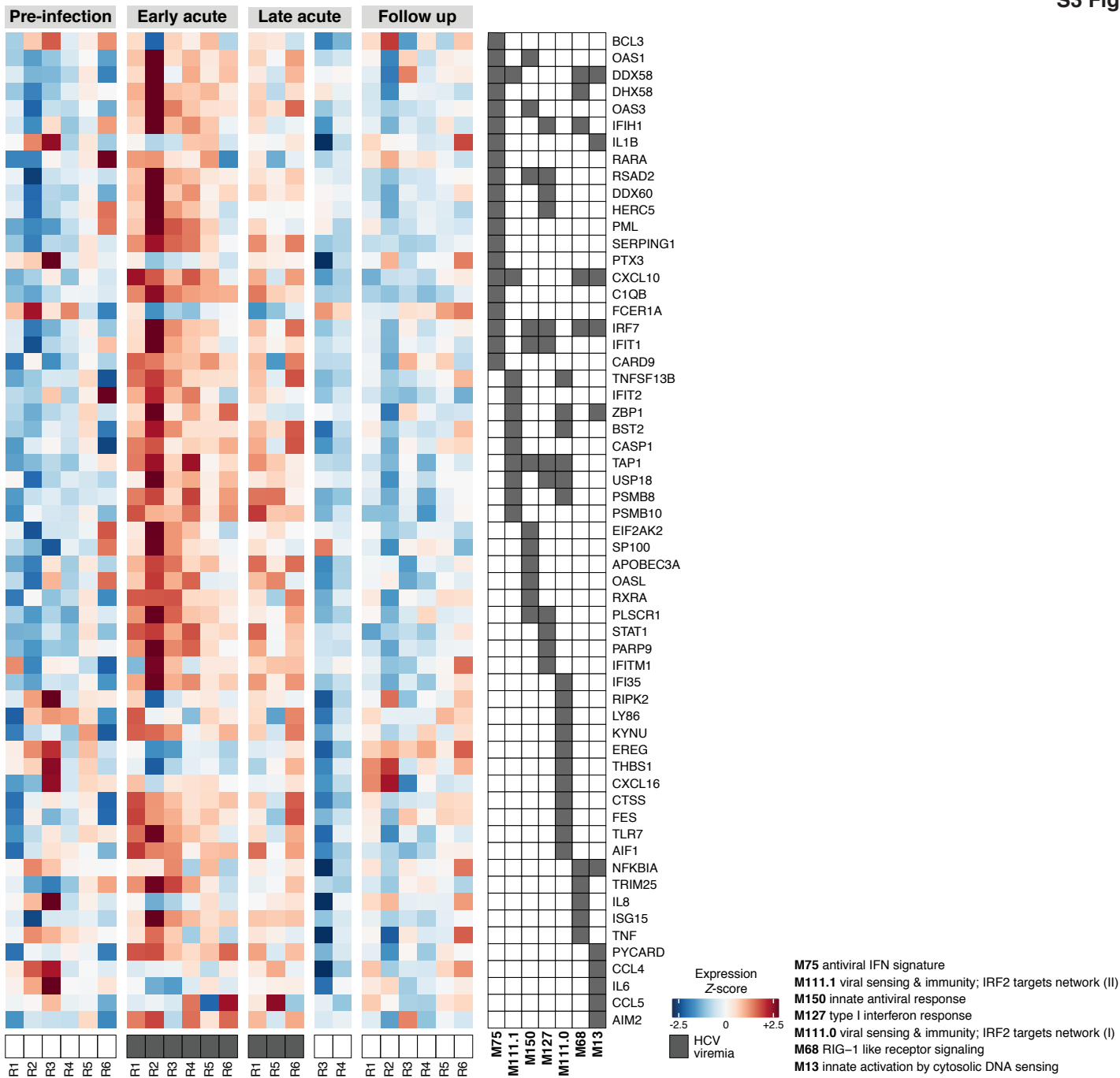

Supplement: S3 Fig — Heatmap displays individual sample scaled expression values (normalized log2 read counts per million, corrected for patient-specific variation, scaled to z-scores by gene) for expressed genes composing enriched BTMs in the INTERFERON/ANTIVIRAL SENSING category. BTM gene membership is denoted in accompanying grid annotation. Dark grey boxes along bottom of heatmap indicate detectable HCV viremia. (PDF) [file ppat.1007290.s003.pdf]

## Vaccine dataset

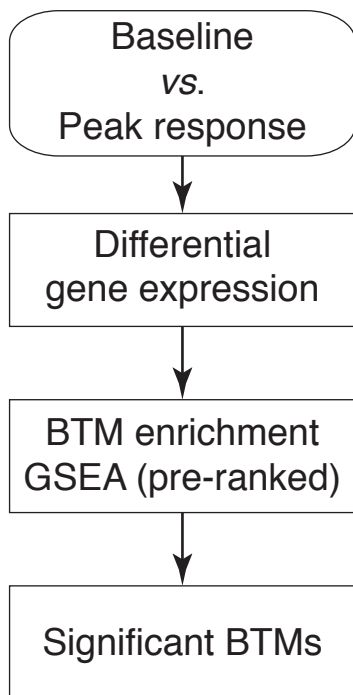

## Acute HCV

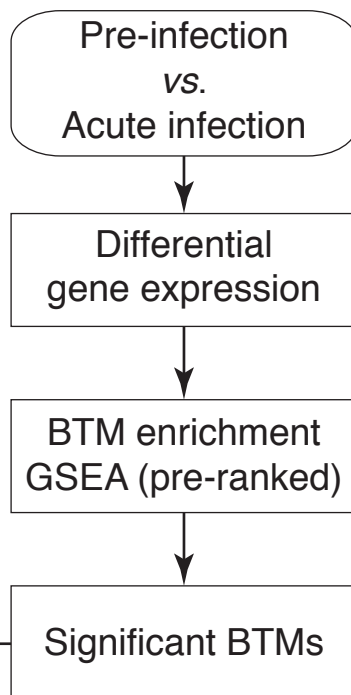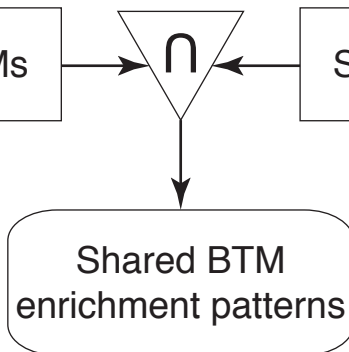

Supplement: S4 Fig — Full details in Materials and methods section. (PDF) [file ppat.1007290.s004.pdf]

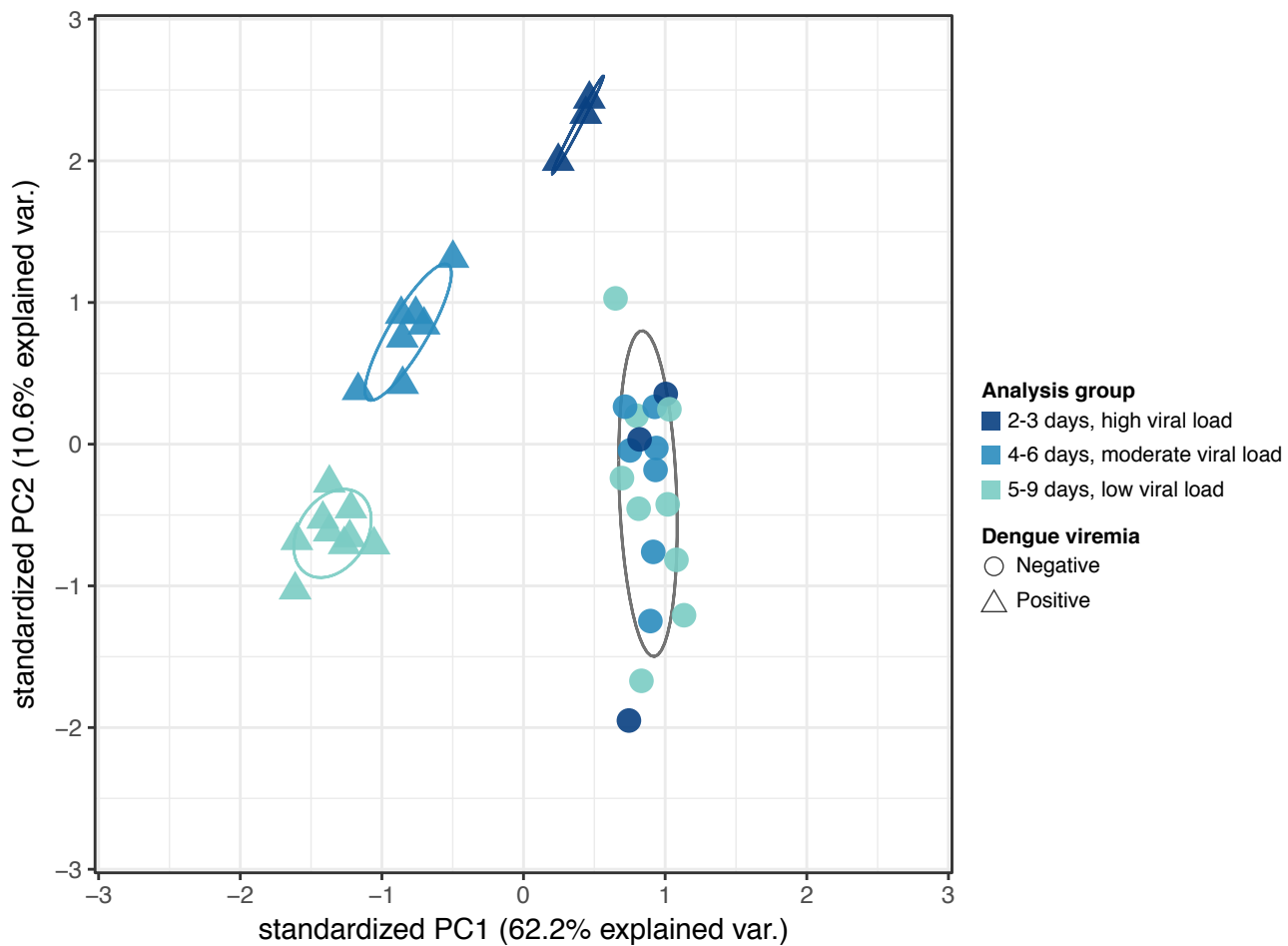

Supplement: S5 Fig — PCA was conducted on all samples with infection and paired convalescence controls from GSE51808 [15]. After correction for sex-specific gene expression variation by the RemoveBatchEffect() function in limma, PCA was performed on the top 1000 most variable genes across all included samples. After assigning sample groupings based on initial PCA, plot colors and ellipses were included to facilitate visualization of group assignments. Ellipses indicate 68% normal probability for each group. (PDF) [file ppat.1007290.s005.pdf]
